# Complicated Relationship between Genetic Mutations and Phenotypic Characteristics in Transient and Permanent Congenital Hypothyroidism: Analysis of Pooled Literature Data

**DOI:** 10.1155/2020/6808517

**Published:** 2020-05-29

**Authors:** Wei Long, Lingna Zhou, Ying Wang, Jiaxuan Liu, Huaiyan Wang, Bin Yu

**Affiliations:** ^1^Department of Medical Genetics, Changzhou Maternal and Child Health Care Hospital Affiliated to Nanjing Medical University, Changzhou 213003, China; ^2^Department of Pediatrics, Changzhou Maternal and Child Health Care Hospital Affiliated to Nanjing Medical University, Changzhou 213003, China; ^3^Department of Epidemiology, Harvard T. H. Chan School of Public Health, Boston, MA 02115, USA

## Abstract

**Purpose:**

Mutations and phenotypic characteristics remain unclear in patients with congenital hypothyroidism (CH), and no study concerning whether the outcome of transient CH (TCH) or permanent CH (PCH) is determined by mutations has been reported.

**Methods:**

We searched the literature up to April 2019. Eligible studies and data extraction were performed. We estimated the relationship between mutations and phenotypic characteristics in pooled patients with CH.

**Results:**

Two hundred forty-one cases were pooled from 41 eligible studies. The thyroid morphology, classification of mutated genes, and types of mutations were different between 94 patients with TCH and 147 patients with PCH. Heterozygous missense mutations prevailed in *PAX8*, *TSHR*, *FOXE1*, and *NKX2-5*, and patients with these mutated genes had a higher risk of PCH (OR = 37.38, 95% CI 5.04–277.21, *P* < 0.001). TCH and PCH have equal shares in patients with mutated *DUOX2* or *DUOXA2*. Dual-site and multisite mutations were frequently detected in *DUOX2*. High phenotypic heterogeneity was observed in mutated *DUOX2* even in the same mutations. However, there was no relationship found between mutations and transient or permanent outcome in patients with mutated *DUOX2*.

**Conclusion:**

Transient or permanent outcomes were influenced by the biological function of mutated genes instead of types of mutations among patients with CH. Patients whose mutations were related to thyroid dysgenesis (TD) were more likely to have PCH. The relationship between mutations and phenotypic characteristics is complicated, and phenotypic characteristics may be affected by mutations and other factors.

## 1. Introduction

Congenital hypothyroidism (CH), the condition of thyroid hormone deficiency present at birth, is the most common neonatal endocrine disorder with an incidence of 1 : 2,000 to 1 : 4,000 live births [1]. Based on the final outcome of thyroid hormone deficiency, CH can be classified into transient CH (TCH) and permanent CH (PCH) [2].

Genetic causes were reported in patients with CH [3, 4]. Thyroid dysgenesis (TD), one of the most common etiologies of CH, was reported to be linked with mutations in genes including thyroid-stimulating hormone receptor (*TSHR*), paired box gene 8 (*PAX8*), thyroid transcription factor 1 (*TTF1*/*NKX2-1*), thyroid transcription factor 2 (*TTF2*/*FOXE1*), and NK2 transcription factor-related locus 5 (*NKX2-5*) [4]. Thyroid dyshormonogenesis (TDH) was reported to be linked with mutations in the thyroid oxidase 2 (*DUOX2*), dual-oxidase maturation factor 2 (*DUOXA2*), thyroglobulin (*TG*), thyroid peroxidase (*TPO*), solute carrier family 5 member 5 (*SLC5A5*), solute carrier family 26 member 4 (*SLC26A4*), and iodotyrosine deiodinase (*IYD*) genes [5, 6]. It has been reported that TD or TDH can cause permanent or transient CH in unclear proportions [1]. However, the relationship between the underlying genotype of TD or TDH and CH outcome remains unclear.

Predicting the outcome of CH for patients is necessary to expeditiously determine whether he or she needs lifelong levothyroxine medication. Some potential indicators, such as the dose of levothyroxine and thyroid hormones at diagnosis or treatment, were studied to predict TCH or PCH [7, 8]. It is well established that the genotype plays an important role in thyroid morphology and the regulation of thyroid hormone levels [4]. However, little is known about whether mutations are related to CH outcomes. Previous case reports or small sample size studies reported patients with various mutations, while it is difficult to assess the relationship between genotype and phenotype resulting from the small sample size [9]. In the present study, we pooled the reported cases of the literature and performed an analysis, to examine (1) the characteristics of gene mutations in TCH and PCH populations and (2) the relationship between mutations and the outcome of PCH or TCH.

## 2. Materials and Methods

### 2.1. Literature Search and Inclusion Criteria

A literature search was performed by MEDLINE (via PubMed) and Web of Science (including Web of Science Core Collection, Chinese Science Citation Database, KCI-Korean Journal Database, MEDLINE, Russian Science Citation Index, and SciELO Citation Index) up to April 2019. The combined terms “congenital hypothyroidism OR thyroid dyshormonogenesis OR thyroid dysgenesis OR goiter OR ectopic OR ectopia” AND “mutation OR mutant OR variation OR variant” AND “permanent OR transient” were searched. Studies that fulfilled the following criteria were included: (1) published original studies, or studies that combined case reports and reviews; (2) clear final outcomes (TCH or PCH) could be inferred; and (3) complete information on the mutation.

### 2.2. Data Extraction

Eligible studies were reviewed by two reviewers, and selected articles were mutually agreed upon by the authors. The following information of the patients was collected: patients' country or region (if not mentioned, the author's region was replaced), sex, blood-spot TSH at newborn screening (bs-TSH), TSH at diagnosis (dTSH), FT3 or FT4 at newborn screening and diagnosis (the thyroid hormones data were not necessary due to the various detection methods and reference ranges), thyroid morphology, mutation information (including gene, position change of nucleotide and amino acid, and monoallelic or biallelic mutation), and clinical outcome (TCH or PCH, based on the thyroid function test results of the authors' reevaluation when patients were over 3 years old [10]).

Studies about gene polymorphisms or patients with aneuploidy, chromosomal structural abnormalities, or undefined copy number variation were not included. For cases studied in multiple publications at different ages, the latest information was included. Repetitive cases in different studies were identified according to the information, including the same authors' name or institution, the same levels of thyroid hormones at newborn screening or diagnosis, and the same mutation. Details of the assessment of CH outcome, the detection method of mutation, and the pathogenicity of mutations in the studies can be found in the original papers.

### 2.3. Statistical Analysis

All statistical analyses were performed by R (version. 3.4.3, http://www.r-project.org). We performed chi-square tests to compare the distribution of demographic and clinical characteristics as well as genetic mutation types between patients with TCH and those with PCH. Univariate analysis was performed to estimate the relationship between mutation and the final outcomes of patients with CH. Two-sided *P* values of less than 0.05 were considered statistically significant.

## 3. Results

### 3.1. Literature Search and Case Inclusion

**Figure 1:**
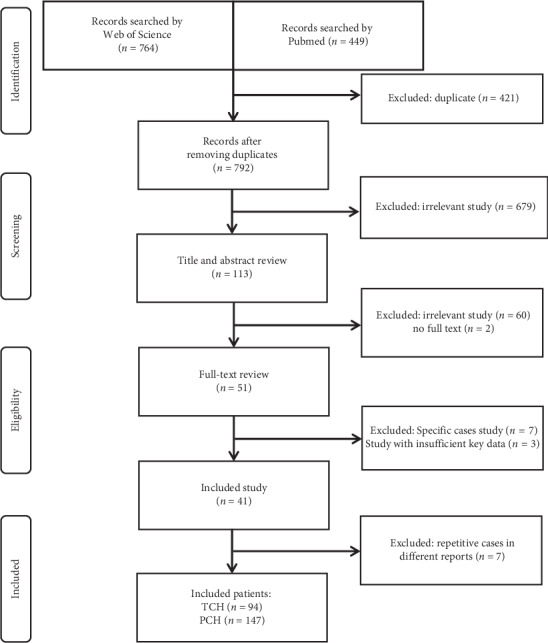
Flow diagram of the present study.

A total of 41 articles fulfilled the inclusion criteria, and 72 family cases and 176 single cases were identified after data extraction (Supplementary Table 1). In the process of data analyses, we identified seven repeat cases in different reports according to the same thyroid hormones and similar authors or authors' institution. Finally, 241 patients with clear final outcomes were identified, including 94 patients with TCH and 147 patients with PCH, and details about included cases are listed in Supplementary Table 1. A flow chart of the article selection process is shown in Figure 1.

### 3.2. Characteristics of Gene Mutations in Patients with CH

According to the data of the included articles, we analyzed the clinical data and mutations. Patients with mutations of *DUOX2*, *DUOXA2*, *TG*, *TPO*, *FOXE1*, *NKX2-5*, *PAX8*, and *TSHR* were identified in our analysis, and *DUOX2* was the most frequent mutation in both patients with TCH and those with PCH. Novel mutations were identified in 34 studies. In 20 of 34 studies, the pathogenicity of novel mutations was evaluated by *in vitro* functional characterization. Eleven other studies evaluated the pathogenicity of novel mutations according to the strategy that mutations were not detected in the healthy control subjects on the basis of *in silico* prediction analysis.

**Figure 2:**
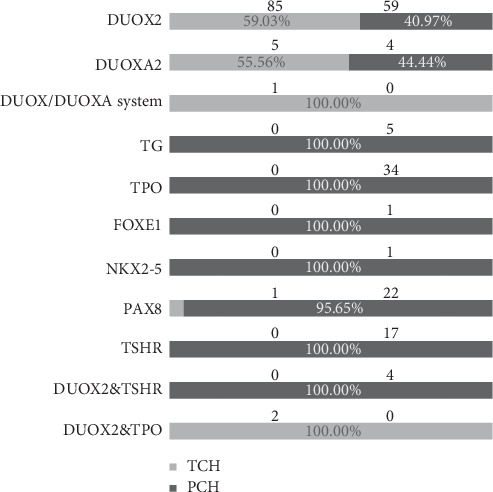
Distribution of mutated genes in 241 included CH cases.

The TCH/PCH proportion of different gene mutations was distinct. Forty-one patients were identified with gene mutation-related TD, including 23 with *PAX8*, 17 with *TSHR*, one with *FOXE1*, and one with *NKX2-5*. One heterozygous *PAX8* mutation patient was TCH, while all of the others were diagnosed with PCH. The result was complicated for genes related to TDH. In our analysis, five *TG* and 34 *TPO* mutation patients were identified, and all of them had PCH, while TCH and PCH had equal shares among the patients with mutations in *DUOX2* or *DUOXA2*. In patients who harbored mutations of two different genes, four patients harboring heterozygous *DUOX2* and *TSHR* were diagnosed with PCH, while two combinational *DUOX2* and *TPO* mutation patients were diagnosed with TCH. In addition, one patient with compound heterozygous c.565T > *C* of *DUOX2* and 43 kB deletion (encompassing *DUOX2*, DUOXA1, and *DUOXA2*) was TCH. The distributions of genotypes in the TCH and PCH groups are shown in Figure 2.

### 3.3. Characteristics of the Patients with TCH and with PCH

We compared the characteristics between patients with TCH and those with PCH, and the results are shown in Table 1. The proportion of area, thyroid morphology, gene classification, and type of mutation were different between patients with TCH and those with PCH, while no difference was observed in sex, mutation state, or location. We also evaluated the relationship between the patient data and the outcome. Compared to normal thyroid morphology, the abnormal morphology of TD, including small volume, hypoplasia, and ectopy, seemed to be a risk predictor of PCH (OR = 21.08, 95% CI: 2.70–164.37, *P*=0.004). Similar results were obtained in the gene classification, and patients with mutations associated with TD were more likely to have PCH than patients with mutations of TDH (OR = 37.38, 95% CI: 5.04–277.21, *P* < 0.001). In terms of the types of mutations, patients with single non-missense mutations, dual-site mutations, or multisite mutations were less likely to have PCH than patients with single missense mutations.

According to the pathogenic mechanism and the distinct TCH/PCH proportion of gene mutations, we further divided genes into three subgroups, *DUOX2*/*DUOXA2*, *TG*/*TPO*, and *PAX8*/*TSHR*/*FOXE1*/*NKX2-5*, and studied the differences in the subgroup of gene classifications (results are shown in Table 2). Most mutations of *PAX8*, *TSHR*, *FOXE1*, and *NKX2-5* were found in patients with PCH except for one *PAX8* mutation in a patient with TCH. For those patients with PCH, heterozygote (78.05%) and single missense mutations (75.61%) prevailed. This characteristic may explain the unusual result that single missense mutations have a higher risk of PCH in all cases. The thyroid morphology of 67.57% of patients was small volume, hypoplasia, or ectopy. All mutations of *TG* and *TPO*, which are associated with TDH, were found in patients with PCH, 86.49% of whom had goiter. Among patients with *DUOX2* mutations, Asian cases were overwhelmingly dominant, and a significant difference was found in the distribution of area between TCH and PCH patients, but no difference was observed in other characteristics. In addition, dual-site and multisite mutations were mainly detected in *DUOX2*/*DUOXA2*, while single mutations were more common in genes associated with TD. We also evaluated the relationship between the mutation data and the final outcome in *DUOX2* mutation patients, but no statistical significance was observed (result is shown in Supplementary Table 2).

### 3.4. Phenotypic Heterogeneity of the *DUOX2* Mutation

In the course of data analysis, we found phenotypic heterogeneity in consanguineous or nonconsanguineous patients with the same *DUOX2* mutation. The different phenotypic cases with the same mutation are summarized in Supplementary Table 3. These mutations included seven missense mutation, one nonsense mutations, one multisite mutation, and one splice site mutation. All cases were reported in East Asia, including China, Korea, and Japan. The different phenotypes were reflected in the final outcome and thyroid morphology. Among those patients, 73.47% were goiter and 66.1% were TCH.

## 4. Discussion

To the best of our knowledge, this is the first study concerning the relationship between mutations and phenotype in previously reported patients with CH. We compared the mutation data between patients with TCH and patients with PCH, and the proportion of genes and mutation types between TCH and PCH was different. The outcome of patients with CH at reevaluation was influenced by the mutated gene. Compared to patients whose mutated gene is related to TDH, patients with mutations related to TD have a higher risk of developing PCH.

Recent evidence points to the possibility of a genetic component in patients with TD [4]. Transcription factors encoded by *PAX8*, *FOXE1*, and *NKX2-5* are necessary for thyroid embryogenesis and are also expressed in other tissues of the developing fetus. Mutations in these genes lead to syndromes combined with TD [11, 12]. It is easy to understand that most patients with CH with mutations in *PAX8*, *FOXE1*, and *NKX2-5* present with PCH and thyroid morphology of dysgenesis in our study. Characteristics of heterozygote or single missense mutations were obvious among those patients. However, some unusual cases were identified. Three PCH cases with normal thyroid but one TCH case with decreased thyroid were reported in the same study, and the authors found that the thyroid gland ranged from aplastic and hypoplastic to normal in patients with the same *PAX8* mutation (p.R31H) and reported the first patient with *PAX8* mutation with an enlarged thyroid gland [13]. TSHR, which is located on the surface of thyroid follicular cells and mediates the effects of TSH, is important for the development and function of the thyroid gland [14]. Although *TSHR* gene mutations were reported to be one of the causes of TD [15] and patients with mutations in *TSHR* all presented with PCH in our study, thyroid morphology was variable. Our results showed that eight of twelve patients with PCH with mutations in *TSHR* have eutopic thyroid glands. Previous studies demonstrated that inactivating *TSHR* mutations results in mild or severe thyroid hypoplasia [16, 17], and the heterogeneity was presumably due to different residual functions of *TSHR* mutations.

We concluded that patients with mutations of *TPO* or *TG* were permanent in our included cases. Both monoallelic and biallelic mutations of *TPO* were classically characterized by total iodide organification defect [18, 19]. Unlike *TPO* or *TG*, equal shares of TCH or PCH were found in the patients with mutations in *DUOX2* or *DUOXA2* in our review, and dual-site and multisite mutations were frequently detected in *DUOX2*. Mutation in *DUOX2*, which is responsible for TDH, was the most frequent mutation in both TCH and PCH patients, especially among Asian populations. Our previous study also demonstrated that *DUOX2* was the most common genetic defect in Chinese patients with CH [20]. Most patients with *DUOX2* pathogenic mutations have a thyroid gland with increased or normal size [21], but Fu et al. [22] reported one female PCH case with decreased thyroid size. In the original study [22], mutations of *DUOX2* were screened by Sanger sequencing, while other genes were not screened. We presumed that a further study of gene test is necessary to interpret this unusual case.

The characteristics of the *DUOX2* mutation and the phenotype-genotype correlations have not yet been fully understood. Our results showed that dual-site or multisite mutations were frequently reported in *DUOX2* but rarely in other genes. We also found that variable phenotypes were caused by *DUOX2* mutations, and there was no relationship between the mutation from *DUOX2* and the outcome of patients with CH at reevaluation, which was controversial in previous studies. Previous studies demonstrated that biallelic *DUOX2* mutations cause severe, permanent hypothyroidism, while monoallelic mutations result in milder, transient hypothyroidism [23, 24]. However, subsequent studies have reported biallelic mutations in *DUOX2* in patients with TCH [25, 26], and monoallelic mutations in patients with PCH were also reported [27, 28]. We found that TCH and PCH have equal shares in patients with *DUOX2* mutations, which demonstrated that monoallelic and biallelic *DUOX2* mutations can both cause transient or permanent CH, while there was no correlation between the final outcome and the mutation type. Although a study showed that patients with one or two *DUOX2* mutations turned out to have subclinical or transient CH and that patients with three or more *DUOX2* mutations were mostly associated with permanent CH [29], another study showed that disease severity, neurodevelopment, and prognosis were not correlated with *DUOX2* mutation types or numbers [30]. Our study confirmed that the number of *DUOX2* mutations did not determine transient or permanent outcomes. It is difficult to predict the outcome by the number of mutations or mutated alleles in patients with *DUOX2* mutations.

Phenotypic heterogeneity with the same *DUOX2* mutation was found in consanguineous or nonconsanguineous patients, implying that the phenotype might be different in individuals with *DUOX2* mutations, even in those with the same mutation. *DUOX2* mutations result in variable phenotypes ranging from mild to severe CH [31], which could be explained by the compensation effect. The coexistence of two DUOX isoforms (DUOX1 and DUOX2) and their corresponding maturation factors could certainly constitute an efficient redundant mechanism to maintain sufficient H_2_O_2_ supply for thyroid hormone synthesis [32]. In addition, phenotypic heterogeneity could be explained by other mechanisms such as unidentified mutations in noncoding regions or other genes, nutrition, environment, or other epigenetic factors [33–36].

The genetic diagnosis of CH could provide great benefits not only to current patients but also in genetic counseling for the next birth or subsequent generations. In addition, it is also helpful to understand the genetic etiology of CH. Our study indicated that the transient or permanent outcome of patients with CH was influenced by the type of genes instead of the form of mutations and patients whose mutations related to TD were more likely to have PCH. However, the limitations of the present study need to be noticed. Although 41 studies were included in this study, the number of CH patients included in the final data analysis was relatively small. This limitation might affect the accurate analysis of relationship between mutations and phenotypes. In addition, it is difficult to predict the outcome by the mutation in patients with the *DUOX2* mutation because of the variable phenotypes resulting from the *DUOX2* mutation. Further study is necessary to understand the complicated relationship between the genetic etiology and phenotypic characteristics of CH.

## 5. Conclusions

The outcome of transient or permanent was influenced by the functional type of mutated genes instead of the form of mutations among patients with CH. Patients whose mutations were related to TD were more likely to have PCH. The relationship between mutations and phenotypic characteristics was complicated, and there was no relationship between mutations and transient or permanent outcome in patients with mutated *DUOX2*.

## Figures and Tables

**Table 1:** Characteristics comparison between the TCH and PCH cases.

	Chi-square test				Univariate analysis^f^		
	TCH (*n* = 94)	PCH (*n* = 147)	Total (*n* = 241)	*P*	OR	95% CI	*P*
Continent				<0.001			
Africa	0 (0.00%)	1 (0.68%)	1		Reference		
Asia	85 (90.43%)	90 (61.22%)	175		§		
Europe^a^	5 (5.32%)	50 (34.01%)	55		§		
South America	4 (4.26%)	6 (4.08%)	10		§		
Sex				0.958			
Male	48 (52.17%)	62 (52.54%)	110		Reference		
Female	44 (47.83%)	56 (47.46%)	100		0.99	0.57–1.70	0.958
Unknown^b^	2	29	31		NA		
Thyroid morphology				<0.001			
Normal	30 (34.48%)	37 (28.46%)	67		Reference		
Goiter/enlarged	56 (64.37%)	67 (51.54%)	123		0.97	0.53–1.76	0.921
Small volume/ectopy	1 (1.15%)	26 (20.00%)	27		21.08	2.70–164.37	0.004
Unknown^b^	7	17	24		NA		
Gene classification^c^				<0.001			
Gene relating to TDH	93 (98.94%)	102 (69.39%)	195		Reference		
Gene relating to TD	1 (1.06%)	41 (27.89%)	42		37.38	5.04–277.21	<0.001
Gene relating to TDH and TD	0 (0.00%)	4 (2.72%)	4		§		
Type of mutation^d^				0.028			
Single missense mutation	18 (19.15%)	53 (36.05%)	71		Reference		
Single non-missense mutation	34 (36.17%)	41 (27.89%)	75		0.41	0.20–0.83	0.013
Dual-site mutations	34 (36.17%)	47 (31.97%)	81		0.47	0.23–0.94	0.033
Multisite mutations	8 (8.51%)	6 (4.08%)	14		0.25	0.08–0.83	0.024
Mutation state^e^				0.419			
Heterozygous	44 (46.81%)	74 (50.34%)	118		Reference		
Homozygous	8 (8.51%)	20 (13.61%)	28		1.49	0.60–3.66	0.388
Compound heterozygous	40 (42.55%)	49 (33.33%)	89		0.73	0.42–1.28	0.267
Combinational heterozygous	2 (2.13%)	4 (2.72%)	6		1.19	0.21–6.76	0.845
Mutation location				0.143			
Monoallelic	44 (46.81%)	83 (56.46%)	127		Reference		
Biallelic	50 (53.19%)	64 (43.54%)	114		0.68	0.40–1.14	0.144

^a^One French-Canadian case with TCH was included in the Europe group. ^b^Cases with unknown sex or thyroid morphology were not included in the comparison of TCH *vs*. PCH. ^c^Gene classification was based on the function of the gene according to previous studies. The TDH genes included *DUOX2*, *DUOXA2*, *TG*, and *TPO*; the TD genes included *FOXE1*, *NKX2-5*, *PAX8*, and *TSHR*; and the TDH and TD genes included *DUOX2* and *TSHR*. ^d^Single non-missense mutations, including single nonsense, frameshift, splice site, or in-frame mutations. One TCH case with a 43 kB pair deletion of chromosome 15 (encompassing *DUOX2*, *DUOXA1*, and *DUOXA2*) was included in the group of multisite mutations. ^e^Compound heterozygous cases harbored at least two mutations of one gene. Combinational heterozygous cases harbored mutations of two different genes. ^f^The odds ratio refers to TCH *vs*. PCH. §The analysis failed because of the small sample size. TCH: transient congenital hypothyroidism; PCH: permanent congenital hypothyroidism; TDH: thyroid dyshormonogenesis; TD: thyroid dysgenesis; NA: not analyzed.

**Table 2:** Characteristics comparison of gene subgroup included cases.

	*DUOX2*/*DUOXA2*		*TG*/*TPO*	*PAX8*/*TSHR*/*FOXE1*/*NKX2-5*		
	TCH (*n* = 90)	PCH (*n* = 63)	PCH (*n* = 39)	TCH (*n* = 1)	PCH (*n* = 41)	*P*_b_
Continent	*P*_a_=0.019					<0.001	
Africa	0	0	0	0 (0.00%)	1 (2.44%)	
Asia	82 (91.11%)	51 (80.95%)	14 (35.90%)	1 (100.00%)	21 (51.22%)	
Europe	4 (4.44%)	11 (17.46%)	24 (61.54%)	0 (0.00%)	15 (36.59%)	
South America	4 (4.44%)	1 (1.59%)	1 (2.56%)	0 (0.00%)	4 (9.76%)	
Sex						0.291
Male	45 (51.14%)	32 (59.26%)	10 (52.63%)	0 (0.00%)	17 (41.46%)	
Female	43 (48.86%)	22 (40.74%)	9 (47.37%)	1 (100.00%)	24 (58.54%)	
Thyroid morphology						<0.001
Normal	28 (33.73%)	17 (32.69%)	5 (13.51%)	0 (0.00%)	11 (29.73%)	
Goiter/enlarged	55 (66.27%)	34 (65.38%)	32 (86.49%)	0 (0.00%)	1 (2.70%)	
Small volume/ectopy	0 (0.00%)	1 (1.92%)	0	1 (100.00%)	25 (67.57%)	
Type of mutation						<0.001
Single missense mutation	18 (20.00%)	17 (26.98%)	5 (12.82%)	0 (0.00%)	31 (75.61%)	
Single non-missense mutation	33 (36.67%)	18 (28.57%)	19 (48.72%)	1 (100.00%)	4 (9.76%)	
Dual-site mutations	34 (37.78%)	23 (36.51%)	15 (38.46%)	0 (0.00%)	6 (14.63%)	
Multisite mutations	5 (5.56%)	5 (7.94%)	0	0	0	
Mutation state						<0.001
Heterozygous	43 (47.78%)	26 (41.27%)	16 (41.03%)	1 (100.00%)	32 (78.05%)	
Homozygous	8 (8.89%)	9 (14.29%)	8 (20.51%)	0 (0.00%)	3 (7.32%)	
Compound heterozygous	39 (43.33%)	28 (44.44%)	15 (38.46%)	0 (0.00%)	6 (14.63%)	
Mutation location						0.001
Monoallelic	43 (47.78%)	28 (44.44%)	21 (53.85%)	1 (100.00%)	32 (78.05%)	
Biallelic	47 (52.22%)	35 (55.56%)	18 (46.15%)	0 (0.00%)	9 (21.95%)	

Combinational heterozygous cases who harbored mutations of two different genes were not analyzed. *P*_a_ was 0.016 in the comparison of area between TCH and PCH in the *DUOX2* subgroup, while *P* > 0.05 was observed in other unmarked comparisons. *P*_b_ was calculated for the characteristics comparison of all patients with CH in the three gene subgroups. TCH: transient congenital hypothyroidism; PCH: permanent congenital hypothyroidism.

## Data Availability

The data used to support the findings of this study are available from the corresponding authors upon request.

## Supplementary Materials

Supplementary Materials Supplementary Table 1: clinical data and mutation of included cases. Note: bsTSH: blood-spot TSH at newborn screening; dTSH: TSH at diagnosis; PCH: permanent CH; TCH: transient CH; fsX: frameshift with an early stop codon. Supplementary Table 2: univariate analysis for mutation data associated with the final outcome in patients with *DUOX2* mutation. Supplementary Table 3: phenotypic heterogeneity in the patients with the same-site mutation of *DUOX2* gene.
