# Supplementary material for: Complicated Relationship between Genetic Mutations and Phenotypic Characteristics in Transient and Permanent Congenital Hypothyroidism: Analysis of Pooled Literature Data
Source: Int J Endocrinol. 2020 May 29;2020:6808517. doi: 10.1155/2020/6808517 (PMC7275948; doi:10.1155/2020/6808517)
Supplement: Supplementary Materials — Supplementary Table 1: clinical data and mutation of included cases. Note: bsTSH: blood-spot TSH at newborn screening; dTSH: TSH at diagnosis; PCH: permanent CH; TCH: transient CH; fsX: frameshift with an early stop codon. Supplementary Table 2: univariate analysis for mutation data associated with the final outcome in patients with DUOX2 mutation. Supplementary Table 3: phenotypic heterogeneity in the patients with the same-site mutation of DUOX2 gene. [file 6808517.f1.zip › 6808517.f1/Supplementary Table2&3.docx]

**Supplementary Tables**

Supplement Table 2. Univariate analysis for mutation data associated with final outcome in patients with *DUOX2* mutation.

|  | Statistics | OR (95%CI) *P* |
| --- | --- | --- |
| Continent |  |  |
| Asia | 124 (86.11%) | Reference |
| Europe ^a^ | 15 (10.42%) | 4.51 (1.36, 14.97) 0.0140 |
| South America | 5 (3.47%) | 0.41 (0.04, 3.78) 0.4309 |
| Sex |  |  |
| Male | 73 (54.89%) | Reference |
| Female | 60 (45.11%) | 0.63 (0.31, 1.28) 0.2020 |
| Thyroid morphology |  |  |
| Normal | 41 (32.54%) | Reference |
| Goiter | 84 (66.67%) | 1.07 (0.49, 2.31) 0.8700 |
| Small volume /ectopy | 1 (0.79%) | § |
| Type of mutation |  |  |
| Single missense mutation | 33 (22.92%) | Reference |
| Single non- missense mutation | 44 (30.56%) | 0.44 (0.17, 1.12) 0.0836 |
| Dual-site mutations | 57 (39.58%) | 0.64 (0.27, 1.51) 0.3056 |
| Multisite mutations | 10 (6.94%) | 0.94 (0.23, 3.88) 0.9331 |
| Mutation state |  |  |
| Heterozygous | 64 (44.44%) | Reference |
| Homozygous | 13 (9.03%) | 1.34 (0.40, 4.44) 0.6353 |
| Compound heterozygous | 67 (46.53%) | 1.12 (0.56, 2.25) 0.7505 |
| Mutation location |  |  |
| Monoallelic | 66 (45.83%) | Reference |
| Biallelic | 78 (54.17%) | 1.00 (0.52, 1.96) 0.9887 |

§The analysis failed because of the small sample size.

Supplement Table 3. Phenotypic heterogeneity in the patients with same site mutation of DUOX2 gene.

| Mutation | Author, year | Case ID | Regions | Sex | bs-TSH | dTSH | Thyroid morphology | Clinical phenotype |
| --- | --- | --- | --- | --- | --- | --- | --- | --- |
| p.[G206V+E879K]+p.H678R | Jin et al., 2014 | 110a | Korea | male | 12.1 | 26.9 | normal | PCH |
|  |  | 111a | Korea | female | 44.7 | 76.8 | goiter | TCH |
| p.H678R | Narumi et al., 2011 | 73 | Japan | male | 75.4 | 103 | normal | PCH* |
|  | Jin et al., 2014 | 99 | Korea | male | 19.1 | 56.4 | normal | TCH |
|  | Jin et al., 2014 | 114 | Korea | male | 44.9 | 23.8 | NA | PCH* |
|  | Jin et al., 2014 | 115 | Korea | male | 30.1 | 153 | NA | PCH* |
|  | Jin et al., 2014 | 116 | Korea | male | 18.5 | 24.3 | NA | PCH |
| p.E879K | C. Fu et al., 2015 | 149 | China | Female | NA | >100 | normal | TCH |
|  | M. Tan et al., 2016  M. Tan et al., 2016  M. Tan et al., 2016  M. Tan et al., 2016 | 186 | China | female | 51.09 | > 75 | goiter | TCH |
|  |  |  | China | female | 9.17 | 13.92 | goiter | **PCH** |
|  |  | 189 | China | male | 48.76 | > 100 | goiter | **PCH** |
|  |  | 211 | China | male | 23.85 | 20.32 | goiter | **PCH** |
| p.A1206T | Wang et al., 2014 | 132 | China | female | 182 | >100 | goiter | PCH |
|  |  | 133 | China | female | 192 | >100 | goiter | **TCH** |
|  |  | 241 | China | female | 141.5 | 186.3 | goiter | **TCH** |
| p.R1334W | Jin et al., 2014 | 113 | Korea | male | 40.8 | 76.8 | goiter | PCH |
|  | C. Fu et al., 2015 | 150 | China | male | NA | >100 | goiter | **TCH** |
|  | M. Tan et al., 2016 | 214 | China | male | 11.76 | > 75 | goiter | **TCH** |
| p.K530X | C. Fu et al., 2015 | 146 | China | female | NA | >100 | goiter | **TCH** |
|  | C. Fu et al., 2015 | 147 | China | male | NA | >100 | goiter | **TCH** |
|  | C. Fu et al., 2015 | 148 | China | female | NA | 47.99 | normal | TCH* |
|  | M. Tan et al., 2016 | 171 | China | male | 14.76 | 86.06 | goiter | TCH* |
|  | M. Tan et al., 2016 | 172 | China | male | 111.45 | > 100 | normal | TCH* |
|  | M. Tan et al., 2016 | 173 | China | female | 20.25 | > 100 | goiter | TCH* |
|  | M. Tan et al., 2016 | 175 | China | female | 122.66 | 23.9 | normal | PCH* |
|  | M. Tan et al., 2016 | 183 | China | male | 14.05 | 9.58 | goiter | **TCH** |
|  | M. Tan et al., 2016 | 190 | China | female | 87.81 | > 75 | goiter | **TCH** |
|  | M. Tan et al., 2016 | 192 | China | female | 81.19 | 24.1 | normal | PCH |
|  | M. Tan et al., 2016 | 193 | China | male | 48.29 | 59.38 | normal | **TCH** |
|  | M. Tan et al., 2016 | 195 | China | male | 24.57 | 21.2 | normal | PCH |
|  | M. Tan et al., 2016 | 197 | China | male | 66.51 | > 100 | goiter | **TCH** |
|  | M. Tan et al., 2016 | 198 | China | female | 19.58 | 93.66 | goiter | **TCH** |
|  | M. Tan et al., 2016 | 199 | China | female | 22.4 | > 100 | goiter | **TCH** |
|  | M. Tan et al., 2016 | 200 | China | male | 52.69 | > 100 | goiter | PCH |
|  | M. Tan et al., 2016 | 204 | China | male | 9.23 | > 100 | goiter | **TCH** |
|  | M. Tan et al., 2016 | 207 | China | male | 13.16 | > 75 | goiter | **TCH** |
|  | M. Tan et al., 2016 | 208 | China | male | 18.51 | 23.76 | goiter | PCH |
|  | M. Tan et al., 2016 | 209 | China | female | 16.12 | > 100 | goiter | **TCH** |
|  | M. Tan et al., 2016 | 210 | China | male | 13.96 | 25.23 | goiter | **TCH** |
|  | M. Tan et al., 2016 | 217 | China | male | 21.94 | > 100 | goiter | **TCH** |
|  | M. Tan et al., 2016 | 220 | China | male | 61.79 | 72.66 | goiter | **TCH** |
|  | Huang et al., 2017 | 231 | China | male | 52.7 | >100 | NA | PCH |
| p.R1110Q | C. Fu et al., 2015 | 145 | China | male | NA | >100 | normal | **TCH** |
|  | M. Tan et al., 2016 | 212 | China | male | 19.72 | 16.1 | goiter | TCH |
|  | M. Tan et al., 2016 | 218 | China | female | 77.45 | 11 | goiter | **PCH** |
|  | M. Tan et al., 2016 | 219 | China | male | 35.29 | > 75 | goiter | **TCH** |
| p.R1334W | Jin et al., 2014 | 113 | Korea | male | 40.8 | 76.8 | goiter | PCH |
|  | C. Fu et al., 2015 | 150 | China | male | NA | >100 | goiter | TCH |
| p.R885Q | Jin et al., 2014 | 96 | Korea | male | 22.1 | 23.2 | normal | TCH |
|  | Jin et al., 2014 | 102 | Korea | male | NA | >200 | NA | **PCH** |
|  | Huang et al., 2017 | 228 | China | female | 22.3 | 86 | normal | **PCH** |
| IVS28+1G>T | M. Tan et al., 2016 | 188 | China | female | 11.62 | > 100 | goiter | **TCH** |
|  |  | 202 | China | female | 18.67 | > 100 | goiter | PCH |
|  |  | 205 | China | male | 27.06 | 42.09 | goiter | **TCH** |
|  |  | 216 | China | female | 42.13 | > 75 | goiter | **TCH** |

* Cases with homozygous mutation. a: case 110 and case 111 were siblings. Highlight clinical phenotype with bold font: dominating phenotype of same mutation. bs-TSH: Blood-spot TSH at newborn screening, dTSH: TSH at diagnosis; NA: Not Available.
